# Effectiveness of a Hydrogen Peroxide Mist (Trophon) System in Inactivating Healthcare Pathogens on Surface and Endocavitary Probes

**DOI:** 10.1017/ice.2016.11

**Published:** 2016-02-04

**Authors:** William A. Rutala, Maria F. Gergen, Emily E. Sickbert-Bennett

**Affiliations:** 1 Hospital Epidemiology, University of North Carolina Health Care, Chapel Hill, North Carolina; 2 Division of Infectious Diseases, UNC School of Medicine, Chapel Hill, North Carolina.

Ultrasound probes are used in sonographic scanning and are commonly used as either surface probes or endocavitary probes. Surface probes are used on intact skin, such as the abdomen; they are considered noncritical and require at least low-level disinfection between patients. Endocavitary probes (eg, transvaginal, transrectal, or transesophageal probes) are considered semicritical because they have direct contact with mucous membranes (eg, vagina, rectum, or pharynx) or non-intact skin. While one could argue that the use of the probe cover changes the category for the endocavitary probe, the Centers for Disease Control and Prevention (CDC) guideline for disinfection and sterilization recommends that a new condom/probe cover should be used for each patient, and because condoms/probe covers and low-level disinfection may fail,^1^ ^,^ ^2^ high-level disinfection of the probe should be performed.^3^ The relevance of this recommendation is reinforced by the finding that sterile ultrasound probe covers and condoms can have a perforation rate from 0% to 81% before and after use.^1^ These studies underscore the need for high-level disinfection of endocavitary probes between examinations.

Ultrasound probes may also be used during surgical procedures and may have contact with sterile body sites. These probes may be covered with a sterile sheath to reduce the level of contamination on the probe and reduce the risk of infection. However, because the sheath does not provide complete protection of the probe, the probes should be sterilized between each patient use, as are other critical items. If this is not possible, then high-level disinfection of the probe should be performed and the probe should be covered with a sterile probe cover.^1^ ^,^ ^3^

Although the most common method of performing high-level disinfection of contaminated endocavitary probes is by immersion in a high-level disinfectant cleared by the Food and Drug Administration (eg, glutaraldehyde), an alternative procedure for disinfecting the endocavitary and surface probes uses a proprietary hydrogen peroxide mist system, which utilizes 35% hydrogen peroxide at 56°C with the probe reaching no more than 40°C (ie, Trophon EPR, Nanosonics, Alexandria, Australia). The effectiveness of this technology, which has been cleared by the Food and Drug Administration for high-level disinfection, is evaluated in this research brief.

This study was conducted at the University of North Carolina (UNC) Hospitals, an 853-bed academic medical center. Trophon EPR and 5 ultrasound probes were loaned to UNC Hospitals for use in the study. In a biological safety cabinet, at least 3 replicates of each probe type (ie, endocavitary or surface probe) were inoculated in 3 specified locations with one of the test organisms: vancomycin-resistant *Enterococcus* [VRE] ATCC #51299, a clinical strain of carbapenem-resistant *Enterobacteriaceae* [CRE] *Klebsiella pneumoniae*, *Clostridium difficile* spores, or *Mycobacterium terrae*. These procedures were fully repeated in a separate experiment to ensure accuracy using the aforementioned defined set of test conditions. The inoculae for VRE, CR-*K. pneumoniae, M. terrae*, and *C. difficile* spores in the presence of 5% fetal calf serum (FCS) produced inoculation levels of 5.59-log_10_, 5.91-log_10_, 5.88-log_10_, and 6.23-log_10_, respectively, and in the absence of 5% FCS they produced inoculation levels of 5.84-log_10_, 6.16-log_10_, 4.89-log_10_, and 6.29-log_10_, respectively. In total, 24 surface probes and 31 endocavitary probes were tested. The presence and resistance of *C. difficile* spores (and not vegetative bacteria) were verified by exposing the stock preparation to dilute hydrochloric acid as specified in the AOAC International sporicidal activity test.^4^

The 15 µL inoculum consisted of trypticase soy broth (TSB; Becton, Dickinson and Company, Sparks, MD) with or without 5% FCS (Remel, Lenexa, KS) as an organic challenge. Each inoculum was spread over an area equal to the size of a dime to prevent pooling of the inoculum. The probes were then allowed to air dry for 30 minutes. After drying, each test probe was processed in the Trophon EPR according to the manufacturer’s instructions. A chemical indicator was used in each cycle to ensure that the critical parameters of the cycle had been met. After processing, the probe was evaluated for surviving microbes by submerging the inoculated portion of the probe in TSB (~400 mL surface, ~1500 mL endocavitary) and shaking it at 80–100 rpm (Fisher Scientific Clinical Rotator, Pittsburgh, PA) for a minimum of 1 hour. After shaking, the TSB for each probe was filtered aseptically (0.2 µ pore size, Thermo Scientific, Waltham, MA), and the filter was removed and placed on media appropriate for each test organism. VRE, CR *Klebsiella pneumoniae*, and *C. difficile* were all plated to sheep blood agar, and *Mycobacterium terrae* was plated to Middlebrook 7H11 Agar. All plates were then incubated at 35–37°C in a manner appropriate for each test organism: aerobically for 48 hours for VRE and *Klebsiella pneumoniae*; anaerobically for 48–96 hours for *C. difficile*; and aerobically for 28 days for *Mycobacterium terrae*.

The results demonstrated complete inactivation (>6-log_10_ reduction) of VRE and a CR-*K. pneumoniae* strain both in the presence and absence of 5% FCS (Table 1). The Trophon EPR system showed good, but not complete, inactivation of *M. terrae* (a 5.2-log_10_ reduction for *M. terrae* with FCS and a 4.6-log_10_ reduction for *M. terrae* without FCS) and *C. difficile* (a 5.1-log_10_ reduction for *C. difficile* spores with FCS and a 6.2-log_10_ reduction for *C. difficile* spores without FCS spores) (Table 1). To simulate a worst-case condition, cleaning was not done prior to disinfection in these experiments, but proper cleaning of probes is necessary to ensure the success of high-level disinfection. Other pathogens that could contaminate vaginal and rectal probes include human papilloma virus.^5^ Published data have demonstrated the activity of Trophon to inactivate HPV^6^ and other pathogens (eg, bacteria, mycobacteria, and viruses), including a >6-log_10_ reduction of *M. terrae* and *C. difficile* spores in carrier tests and a >6-log_10_ reduction in *M. terrae* on inoculated ultrasound probes.^7^ These results differ slightly from those presented here, presumably due to the differences in testing methodology. In our study, only the probe devices were inoculated (ie, carriers of different materials were not tested), and for recovery of bacteria on the probe, the probes were immersed in media (ie, not swabbed,^7^ which would likely result in a lower recovery rate).

**TABLE 1:** Proportion of Surface and Endocavitary Probes Positive After Trophon System Processing According to the Presence or Absence of an Organic Challenge*

5% Fetal Calf Serum-FCS	Probes with vancomycin-Resistant *Enterococcus* (VRE), No./Total	Probes with CR *Klebsiella pneumoniae*, No./Total	Probes with *Mycobacterium terrae*, No./Total (mean log_10_ reduction and 95% CI)	Probes with *Clostridium difficile* spores, No./Total (mean log_10_ reductions and 95% CI)
Present	0/7	0/6	4/9 (5.19 [4.61–5.76])	3/6 (5.12 [4.42–5.83])
Absent	0/6	0/6	1/6 (4.62 [4.07–5.17])	1/9 (6.23 [6.02–6.43])

* The inoculum control was handled like the test but not processed in the Trophon. Numbers represent a proportion of the number of probes positive with the test organisms per number of probes tested. No statistical difference in the proportion of positive probes by test organism with and without fetal calf serum (Fisher’s exact test; *P*>.05).

The Trophon system processes the portion of the probe that has contact not only with mucous membrane but also with the handle of an endocavitary probes, which also may be contaminated.^8^ It is an alternative to high-level chemical disinfection for ultrasound probes.
